# Comparative outcomes of COVID-19 in patients with primary Sjögren’s syndrome treated with hydroxychloroquine versus methotrexate: a retrospective cohort study

**DOI:** 10.1080/07853890.2025.2527361

**Published:** 2025-07-06

**Authors:** Jih-Jin Tsai, Li-Teh Liu, Pei-Lun Liao, James Cheng-Chung Wei

**Affiliations:** a Tropical Medicine Center, Kaohsiung Medical University Hospital, Kaohsiung, Taiwan; b Division of Infectious Diseases, Department of Internal Medicine, Kaohsiung Medical University Hospital, Kaohsiung, Taiwan; c School of Medicine, College of Medicine, Kaohsiung Medical University, Kaohsiung, Taiwan; d Department of Medical Laboratory Science and Biotechnology, College of Medical Technology, Chung Hwa University of Medical Technology, Tainan, Taiwan; e Center for Health Data Science, Department of Medical Research, Chung Shan Medical University Hospital, Taichung, Taiwan; f Institute of Medicine, Chung Shan Medical University, Taichung, Taiwan; g Department of Allergy, Immunology & Rheumatology, Chung Shan Medical University Hospital, Taichung, Taiwan; h Department of Nursing, Chung Shan Medical University, Taichung, Taiwan; i Graduate Institute of Integrated Medicine, China Medical University, Taichung, Taiwan

**Keywords:** COVID-19, COVID-19 outcome, hydroxychloroquine, methotrexate, primary Sjögren’s syndrome, SARS-CoV-2 infection

## Abstract

**Background:**

People with rheumatic diseases are considered at risk for severe coronavirus disease 2019 (COVID-19) because of their underlying abnormal immune responses and frequent disease-modifying antirheumatic drug (DMARD) use. We aimed to explore COVID-19 outcomes in patients with primary Sjögren’s syndrome (pSS) receiving different conventional synthetic DMARDs.

**Methods:**

The study was a retrospective analysis of real data from the TriNetX platform between 1 January 2020 and 30 June 2023. We compared COVID-19 outcomes between cohorts of pSS patients treated with hydroxychloroquine (HCQ) and methotrexate (MTX).

**Results:**

After propensity score matching, 1045 and 1045 pSS patients in the HCQ and MTX cohorts were included in the analysis. Our study revealed that the risk of COVID-19 in the HCQ cohort was significantly 25.9% lower than that in the MTX cohort. Among 18–64-year-old patients and unvaccinated patients, those in the HCQ cohort had a significantly lower risk of contracting COVID-19 than did those in the MTX cohort. Regarding the comorbidities of chronic kidney disease and neoplasms, the HCQ cohort had a significantly lower risk of adverse outcomes than the MTX cohort.

**Conclusions:**

This is the first study in which a significant number of pSS patients was enrolled and head-to-head to compare the risk of COVID-19 incidence and adverse outcomes between those receiving HCQ and those receiving MTX, and we concluded that the risk of COVID-19 incidence was significantly lower in the HCQ cohort than in the MTX cohort. However, there were no significant differences in adverse COVID-19 outcomes between the cohorts.

## Introduction

Coronavirus disease 2019 (COVID-19), caused by severe acute respiratory syndrome coronavirus-2 (SARS-CoV-2), has had a great impact and caused challenges worldwide. The clinical spectrum of COVID-19 ranges from no symptoms to critical illness [1]. Age, race, underlying medical conditions, and immunosuppressant use are associated with a greater risk of severe COVID-19 [2,3].

Sjögren syndrome (SS) includes primary Sjögren syndrome (pSS) according to 2016 American College of Rheumatology/European League Against Rheumatism (ACR/EULAR) classification criteria [4], which occurs in the absence of another underlying rheumatic disorder, and secondary SS, which is associated with another underlying rheumatic disease, such as systemic lupus erythematosus (SLE) [5], rheumatoid arthritis (RA), or systemic scleroderma.

pSS is a systemic autoimmune disease that is overwhelmingly diagnosed in women (>95%) aged between 30 and 60 years in two-thirds of cases [6]. The key clinical feature of pSS is the development of sicca symptoms, which is reported by >95% of patients and is accompanied by a wide variety of systemic manifestations, including autoimmune damage to internal organs, in a significant number of patients [7,8]. pSS is not rare, affecting approximately one in every 400 people [9].

SARS-CoV-2 infection may be associated with an exaggerated immune response driven by interleukin-6 (IL-6), tumor necrosis factor-α, and cytokine storms, which have been identified as the key contributors to the development of severe disease [10,11]. Dysregulation of the innate immune barrier plays a pivotal role in pSS pathogenesis, especially in the early phases of the disease, through a mechanism involving the interferon pathway. On the other hand, persistent B-cell activation and the proliferation of Th1 and Th17 cells contribute to disease progression. Cytokine inhibition (with tocilizumab, an IL-6 inhibitor) is effective against both COVID-19 and pSS. The complex interplay between COVID-19 and pSS deserves more attention, and the associated studies thus far have not reached a consistent conclusion [12].

People with rheumatic and systemic autoimmune diseases are considered at risk for severe COVID-19 because of their underlying abnormal immune responses and frequent use of immunosuppressive drugs. Conventional synthetic disease-modifying antirheumatic drugs (csDMARDs) and biologic DMARDs (bDMARDs) [13] are used to manage both glandular and systemic (extraglandular) manifestations in the treatment of pSS patients [14]. Hydroxychloroquine(HCQ) and methotrexate (MTX) are the most commonly used csDMARDs [15]. Recent research has focused on COVID-19 outcomes in patients with RA and SLE, given their frequent use of immunosuppressive therapies and altered immune responses. Studies in RA and SLE cohorts have shown that certain DMARDs, including methotrexate, may be associated with increased susceptibility to infections and potentially worse COVID-19 outcomes, while hydroxychloroquine has not demonstrated consistent protective effects against SARS-CoV-2 infection or severe disease in these populations [16–18]. Despite these insights, the risk and outcomes of COVID-19 in patients with primary Sjögren’s syndrome (pSS) remain largely unexplored. pSS differs from RA and SLE in its pathogenesis, clinical manifestations, and immunological profile, highlighting the need for disease-specific data to inform management decisions during the pandemic.

HCQ and MTX are the most commonly prescribed csDMARDs for pSS. Beyond their widespread use, these agents have distinct immunomodulatory mechanisms: HCQ interferes with endosomal acidification and toll-like receptor (TLR) signaling [13], which may affect viral entry and replication, while MTX primarily acts as an anti-metabolite and immunosuppressant, potentially increasing infection risk [18]. The differential effects of these drugs on viral infections and immune responses have been the subject of debate, especially during the COVID-19 pandemic. The effects of previous exposure to HCQ and MTX in rheumatic disease cohorts on COVID-19 incidence and outcomes have been reported; however, no head-to-head studies have directly compared the impact of HCQ versus MTX on COVID-19 risk and outcomes specifically in pSS patients [16–19].

Addressing this gap is crucial for optimizing therapeutic strategies in this unique patient population. Therefore, our objective was to compare and analyze the incidence and adverse outcomes of COVID-19 in pSS patients treated with HCQ and MTX through the TriNetX platform.

## Materials and methods

### Study design and data sources

This was a retrospective cohort analysis utilizing data aggregated from TriNetX, which comprises real-world data within the life sciences and healthcare domains. TriNetX encompasses the deidentified electronic medical records (EMRs) of more than 250 million individuals sourced from more than 120 global healthcare organizations (HCOs). Further information about TriNetX can be accessed on the website: https://trinetx.com/?mc_cid=7e2ecd5bc5&mc_eid=%5BUNIQID%5D. TriNetX employs a standardized framework to ensure data quality and encompasses three primary categories of quality metrics: conformance, completeness, and plausibility [20]. TriNetX has been utilized to execute numerous high-quality studies [21,22].

The data and analysis were conducted in February 2024. We utilized the U.S. Collaborative Network, a subnet of the TriNetX platform, for the relevant analyses. This network comprises 60 HCOs. In line with our study objectives, we limited the study period to encompass dates between 1 January 2020 and 30 June 2023 and built a cohort of more than 57 million participants.

### Study subjects

SS patients were identified by the International Statistical Classification of Diseases, Tenth Revision, Clinical Modification (ICD-10-CM) code M35.0. The study subjects included SS [ICD-10-CM code = M35.0] patients (aged ≥ 19 years old) enrolled in the TriNetX database. Individuals eligible for this study were SS patients who visited at least twice and received HCQ or MTX at the same time during the study period. We categorized the patients in the SS cohort into two groups: those who received HCQ and those who received MTX for at least 14 days (as the index date) before they developed COVID-19. To clarify the effects of the medications of interest on patients with SS, we excluded patients with secondary SS, including erythematosus (SLE) [ICD-10-CM code = M32], RA [ICD-10-CM codes = M05-06], scleroderma [ICD-10-CM codes = L90.0, L94.0, L94.1, and L94.3], systemic sclerosis [ICD-10-CM code = M34], and dermatomyositis (dermatomyositis or polymyositis) [ICD-10-CM codes = M36.0 and M33]. We then excluded SS patients who had COVID-19 before the index date (Supplementary Table 1: Coding in this study).

### Ethics statement

The TriNetX platform adheres to the Health Insurance Portability and Accountability Act (HIPAA) and the General Data Protection Regulation (GDPR). The Western Institutional Review Board (WIRB) granted TriNetX a waiver because it solely aggregates counts and statistical summaries of deidentified information. Furthermore, the use of TriNetX in this study was approved by the Institutional Review Board of Chung Shan Medical University Hospital (CSMUH No: CS2-21176).

### Outcomes

The outcomes of interest included the following:

#### The incidence of COVID-19

COVID-19 was defined as a positive SARS-CoV-2-related RNA test result (TNX code:9088, LOINC: 94309-2, 94316-7, 94500-6, 94502-2, 94533-7, 94534-5, 94559-2, 94565-9, 94758-0, 94759-8, 94845-5, 95406-5, 95409-9, 95608-6, 96763-8, 94760-6) or a related diagnosis defined by the ICD-10-CM code (U07.1, B34.2, J12.82, B97.29, Z86.16).

#### Medical utilization

Medical utilization was defined as hospitalization, inpatient encounter, critical care services, or mechanical ventilation. Codes included: for hospitalization, CPT codes 1013659, 1013699, 1013729, or inpatient encounter; for critical care, CPT code 1013729; for mechanical ventilation, ICD-10-CM codes 5A1935Z, 5A1945Z, 5A1955Z, 0BH17EZ, 0BH18EZ, 0BH13EZ, ICD-9-CM code 39.65, or CPT codes 31500, 1015098, and 1022227. These codes identify various medical interventions received by patients.

##### Mortality

Mortality status was defined as a deceased status.

##### Adverse outcomes: combined with medical utilization and mortality outcomes

We used a 14-day washout period after the index date for measuring outcomes to prevent reverse causality. All outcomes that occurred 14 days after the index event were included.

### Covariates

To mitigate potential confounding effects, the present study included the following covariate factors, assessed within 1 year prior to the index date:

***Demographic*** variables, including age at the index date, race (categorized as white, black or African American, Asian, American Indian or Alaskan Native, other race or unknown), and socioeconomic status, were represented by proxy codes (ICD-10-CM codesZ55-Z65).

***Lifestyle*** factors included tobacco use (ICD-10-CM codeZ72.0, serving as a proxy for smoking), nicotine dependence (ICD-10-CM codeF17, serving as a proxy for smoking), and alcohol-related disorders (ICD-10-CM codeF10, serving as a proxy for alcohol consumption). To ensure a balanced health status and ***medical utilization*** between the cohorts, we also included office or other outpatient services (defined by CPT code 1013626), emergency department services (CPT code 1013711), and hospital inpatient services (CPT code 1013659).

All ***comorbidities*** were defined using ICD-10-CM codes. The comorbidities considered in this study included neoplasms (C00-D49), hypertensive diseases (I10-I16), cerebrovascular diseases (I60-I69), diabetes mellitus (E08-E13), hyperlipidemia (E78), chronic lower respiratory diseases (J40–J4A), chronic kidney disease (CKD) (N18), noninfective enteritis and colitis (K50–K52), diseases of the liver (K70–K77), sleep disorders (G47), depression (F32) and ischemic heart disease (I20–I25).

The utilization of ***medications*** was determined using the anatomical therapeutic chemical (ATC) codes. We included the following medications for analysis: nonsteroidal anti-inflammatory drugs (NSAIDs; ATC code M01A), corticosteroids for systemic use (H02), abatacept (L04AA24), rituximab (L01FA01), sulfasalazine (A07EC01), minocycline (J01AA08), cyclophosphamide (L01AA01), leflunomide (L04AA13), azathioprine (L04AX01), cyclosporine (L04AA01), belimumab (1092437), tocilizumab (612865) and ianalumab (OMOP5181464).

### Statistical analyses

To mitigate the influence of confounding factors, we employed TriNetX’s built-in capability to generate propensity scores and conducted 1:1 matching using greedy nearest neighbor matching, as detailed at https://support.trinetx.com/hc/en-us/articles/360011978033-In-compare-outcomes-how-are-patients-matched-when-balancing-cohorts/. A caliper of 0.1 pooled standard deviations of the two groups was employed during the matching process for variables such as age at index, sex, race, socioeconomic status, lifestyle factors, comorbidities and NSAIDs. We assessed the comparability between the two groups before and after matching using standardized mean differences (SMDs). An SMD less than 0.1 indicated that both cohorts were well balanced.

We utilized Kaplan–Meier analysis to estimate the probability of the outcomes. The hazard ratios (HRs), associated confidence intervals (CIs), and test for proportionality were computed using R’s Survival package v3.2–3, as outlined at https://support.trinetx.com/hc/en-us/articles/360053133594-How-does-TriNetX-test-for-proportionality-on-a-hazard-ratio-. The log-rank test was used to determine whether there were differences in survival curves between the cohorts and was conducted within the TriNetX platform.

Additionally, we performed five subgroup analyses to investigate variations between the cohorts, including sex, age at index (18–64 years/≧65 years), race (white/black or African American or others), comorbidity (diabetes mellitus, CKD, chronic lower respiratory diseases, cerebrovascular diseases, and neoplasms) and medications used (corticosteroids).

Two sensitivity tests were also conducted. First, we aimed to explore the impact of vaccination in the two groups of pSS patients without COVID-19 on the incidence of COVID-19 and adverse outcomes. Second, we modified the definition of the index date for pSS patients receiving HCQ or MTX for 3 months to 3 years and then analyzed the outcomes.

## Results

### Characteristics of the study subjects

**Figure 1.**
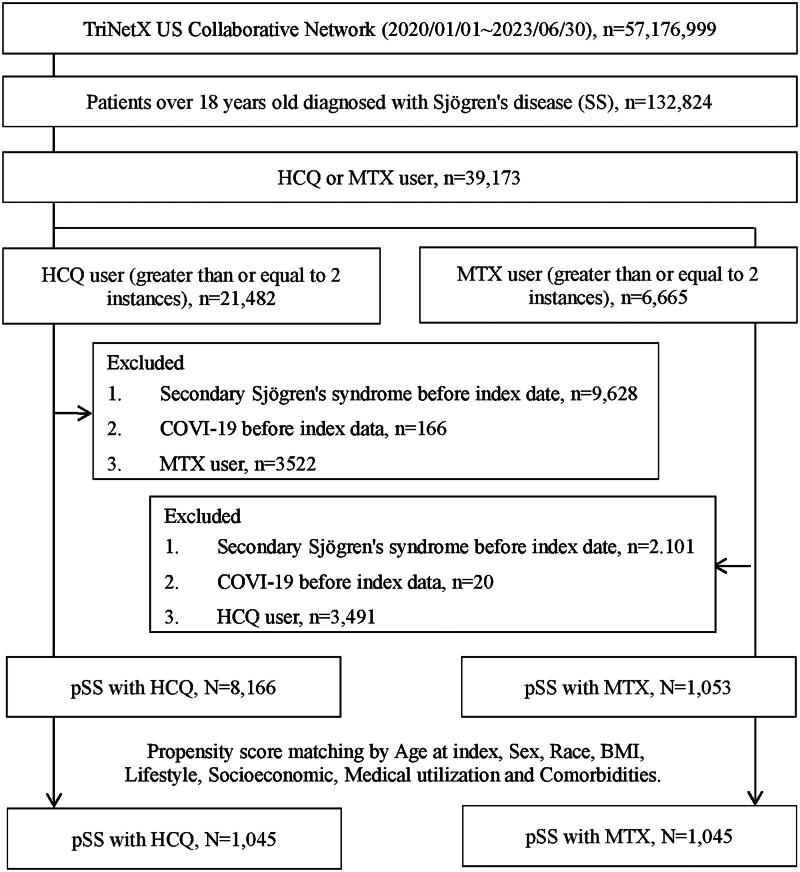
Selection process.

After propensity score matching, 1045 patients were included in the pSS cohort treated with HCQ, and an equal number of patients were included in the pSS cohort treated with MTX. The selection process is visually depicted in Figure 1.

The study subjects’ baseline characteristics are displayed in Table 1, both before and after the matching process. Propensity score matching was performed for age at index, sex, race, socioeconomic status, lifestyle factors, comorbidities, and use of NSAIDs. Nevertheless, after matching, these discrepancies were minimized and fell within an acceptable range, with SMDs of less than 0.1 for the matched variables. According to TriNetX’s policy, patient counts of ≤10 are automatically displayed as “10” to prevent potential re-identification of individuals. This is a standard obfuscation policy implemented by TriNetX to comply with privacy regulations and institutional review board (IRB) standards for the protection of protected health information (PHI). In large-scale analyses or when the affected cell represents a very small proportion of the total population, the rounding has negligible influence on the main outcomes and conclusions.

**Table 1.** Baseline characteristics of the study subjects (before and after matching).

	Before PSM			After PSM		
	pSS cohort treated with HCQ	pSS cohort treated with MTX	SMD	pSS cohort treated with HCQ	pSS cohort treated with MTX	SMD
***N***	8,166	1,053		1,045	1,045	
**Age at index** (mean ± SD)	53.5 ± 15.1	56.7 ± 15.0	**0.211**	56.8 ± 14.3	56.7 ± 15.0	0.011
**Sex**						
Female	7191 (88.1%)	853 (81%)	0.196	851 (81.4%)	852 (81.5%)	0.002
Male	554 (6.8%)	163 (15.5%)	**0.279**	157 (15%)	156 (14.9%)	0.003
**Ethnicity**						
Hispanic or Latino	536 (6.6%)	85 (8.1%)	0.058	92 (8.8%)	84 (8%)	0.028
Not Hispanic or Latino	5797 (71%)	791 (75.1%)	0.093	779 (74.5%)	784 (75%)	0.011
Unknown ethnicity	1833 (22.4%)	177 (16.8%)	**0.142**	174 (16.7%)	177 (16.9%)	0.008
**Race**						
American Indian or Alaskan Native	44 (0.5%)	10 (0.9%)	0.048	0 (0%)	10 (1%)	**0.139**
Asian	270 (3.3%)	33 (3.1%)	0.01	27 (2.6%)	33 (3.2%)	0.034
Black or African American	829 (10.2%)	118 (11.2%)	0.034	118 (11.3%)	118 (11.3%)	<0.001
White	5696 (69.8%)	753 (71.5%)	0.039	760 (72.7%)	746 (71.4%)	0.03
Other Race	241 (3%)	42 (4%)	0.057	33 (3.2%)	41 (3.9%)	0.041
**Lifestyle factors**						
Tobacco use	294 (3.6%)	44 (4.2%)	0.03	41 (3.9%)	44 (4.2%)	0.015
Nicotine dependence	314 (3.8%)	46 (4.4%)	0.026	39 (3.7%)	46 (4.4%)	0.034
Alcohol-related disorders	103 (1.3%)	13 (1.2%)	0.002	15 (1.4%)	13 (1.2%)	0.017
**Socioeconomic and psychosocial circumstances**	227 (2.8%)	25 (2.4%)	0.026	23 (2.2%)	25 (2.4%)	0.013
**Medical utilization**						
Hospital inpatient services	303 (3.7%)	65 (6.2%)	**0.114**	59 (5.6%)	59 (5.6%)	<0.001
Emergency department services	841 (10.3%)	117 (11.1%)	0.026	106 (10.1%)	115 (11%)	0.028
Office or other outpatient services	4731 (57.9%)	611 (58%)	0.002	585 (56%)	606 (58%)	0.041
**Comorbidities**						
Hypertensive diseases	1710 (20.9%)	248 (23.6%)	0.063	228 (21.8%)	241 (23.1%)	0.03
Diabetes mellitus	581 (7.1%)	124 (11.8%)	**0.16**	113 (10.8%)	120 (11.5%)	0.021
Hyperlipidemia	1346 (16.5%)	217 (20.6%)	**0.106**	208 (19.9%)	212 (20.3%)	0.01
Chronic kidney disease (CKD)	344 (4.2%)	33 (3.1%)	0.057	31 (3%)	33 (3.2%)	0.011
Chronic lower respiratory diseases	995 (12.2%)	118 (11.2%)	0.03	108 (10.3%)	117 (11.2%)	0.028
Cerebrovascular diseases	298 (3.6%)	42 (4%)	0.018	32 (3.1%)	41 (3.9%)	0.047
Neoplasms	1312 (16.1%)	226 (21.5%)	**0.139**	198 (18.9%)	219 (21%)	0.05
Noninfective enteritis and colitis	251 (3.1%)	50 (4.7%)	0.086	39 (3.7%)	49 (4.7%)	0.048
Diseases of liver	450 (5.5%)	39 (3.7%)	0.086	34 (3.3%)	39 (3.7%)	0.026
Sleep disorders	1020 (12.5%)	150 (14.2%)	0.052	146 (14%)	148 (14.2%)	0.006
Ischemic heart diseases	410 (5%)	55 (5.2%)	0.009	47 (4.5%)	54 (5.2%)	0.031
Depressive episode	958 (11.7%)	116 (11%)	0.023	110 (10.5%)	113 (10.8%)	0.009
**Medication**						
NSAIDs	2369 (29%)	306 (29.1%)	0.001	305 (29.2%)	302 (28.9%)	0.006
Corticosteroids for systemic use	3134 (38.4%)	561 (53.3%)	**0.302**	427 (40.9%)	553 (52.9%)	**0.243**
Other DMARDs						
Abatacept	12 (0.1%)	10 (0.9%)	**0.109**	10 (1%)	10 (1%)	<0.001
Rituximab	42 (0.5%)	24 (2.3%)	**0.151**	10 (1%)	24 (2.3%)	**0.106**
Sulfasalazine	46 (0.6%)	16 (1.5%)	0.094	10 (1%)	16 (1.5%)	0.052
Minocycline	41 (0.5%)	10 (0.9%)	0.053	12 (1.1%)	10 (1%)	0.019
Cyclophosphamide	10 (0.1%)	12 (1.1%)	**0.129**	10 (1%)	11 (1.1%)	0.01
Leflunomide	44 (0.5%)	15 (1.4%)	0.09	10 (1%)	15 (1.4%)	0.044
Azathioprine	106 (1.3%)	12 (1.1%)	0.014	14 (1.3%)	12 (1.1%)	0.017
Cyclosporine	544 (6.7%)	64 (6.1%)	0.024	76 (7.3%)	63 (6%)	0.05
Belimumab	10 (0.1%)	10 (0.9%)	**0.113**	10 (1%)	10 (1%)	<0.001
Tocilizumab	10 (0.1%)	10 (0.9%)	**0.113**	10 (1%)	10 (1%)	<0.001
Ianalumab	0 (0%)	0 (0%)	–	10 (1%)	10 (1%)	<0.001

Bold font indicates that the standardized difference was greater than 0.1. If the number of patients is less than or equal to 10, the results are shown as a count of 10.

pSS: primary Sjogren syndrome; HCQ: hydroxychloroquine. MTX: methotrexate. SMD: standardized mean difference; SD: standard deviation. NSAIDs: nonsteroidal anti-inflammatory drugs; DMARDs: disease-modifying antirheumatic drugs.

*Propensity score matching (PSM) was performed on age at the index date, sex, race, socioeconomic status, lifestyle factors, comorbidities, and use of NSAIDs.

### pSS patients have lower risk of COVID-19 infection using hydroxychloroquine

**Figure 2.**
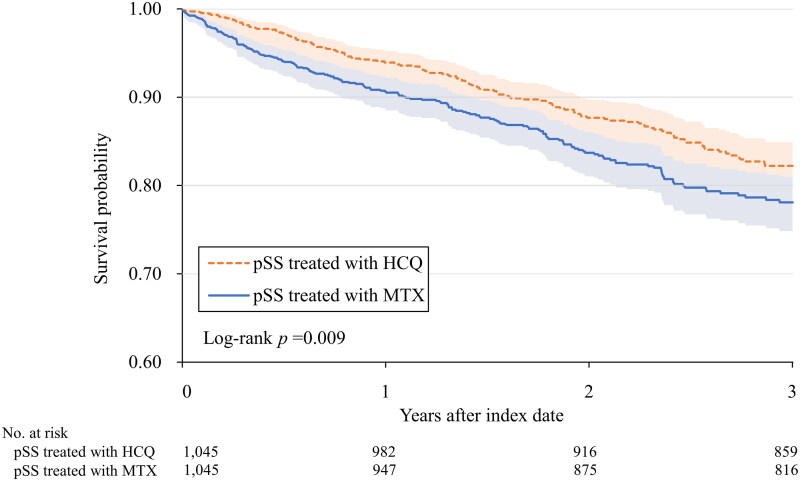
Kaplan–Meier curves for the cumulative survival probability (%) of contracting COVID-19.

Table 2 presents the number of patients who were infected with COVID-19 in both cohorts, accompanied by the 3-year adjusted HRs for the pSS cohort treated with HCQ compared to the pSS cohort treated with MTX. There was a significantly lower risk of COVID-19 (positive laboratory test result or ICD-10-CM code) in the pSS cohort treated with HCQ (HR:0.741, 95% CI:0.592-0.929) than in the pSS cohort treated with MTX. Kaplan–Meier curves for the cumulative probability (%) of COVID-19 are shown in Figure 2, and the log-rank test yielded a *p* of 0.009.

**Table 2.** Risk of contracting COVID-19.

Outcome	Patients with outcome	Cumulative survival probability (%)				Hazard ratio* (95% CI)
		6 months	12 months	24 months	36 months	
Incidence of COVID-19						
COVID-19 (laboratory test result: positive)						
pSS treated with HCQ	49	98.92	97.21	95.87	93.53	0.791 (0.543, 1.153)
pSS treated with MTX	61	98.54	97.38	94.10	91.98	Reference
COVID-19 (ICD-10-CM code)						
pSS treated with HCQ	121	97.54	94.76	88.91	83.90	**0.741 (0.585, 0.940)**
pSS treated with MTX	157	94.27	91.12	85.29	80.39	Reference
COVID-19 (positive result or ICD-10-CM code)						
pSS treated with HCQ	134	97.24	93.93	87.66	82.22	**0.741 (0.592, 0.929)**
pSS treated with MTX	174	94.08	90.62	83.70	78.10	Reference

*Note*. Bold font indicates statistical significance.

*Hazard ratio for outcomes among Sjögren’s syndrome patients in the HCQ group compared to Sjögren’s syndrome patients in the MTX group (after propensity score matching); *Propensity score matching (PSM) was performed on age at the index date, sex, race, socioeconomic status, lifestyle factors, comorbidities, and use of NSAIDs; *95% CI 95% confidence interval.

### Risk of adverse COVID-19 outcomes using hydroxychloroquine compared to methotrexate in patients with pSS

**Figure 3.**
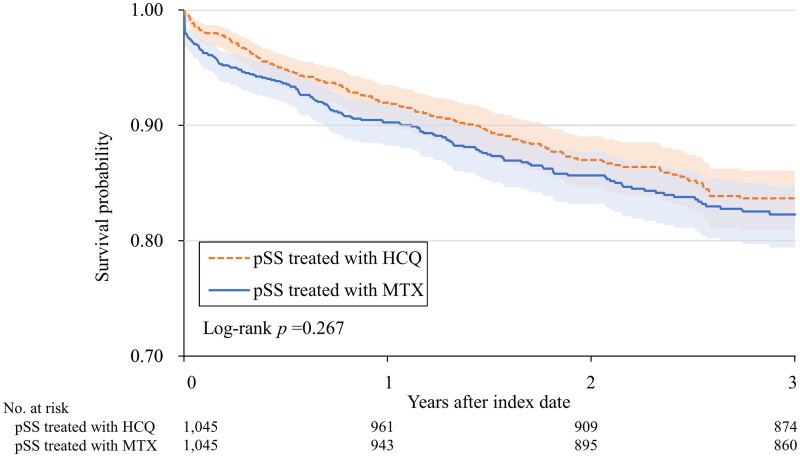
Kaplan–Meier curves for the cumulative survival probability (%) of adverse outcomes.

Table 3 illustrates the risk for adverse outcomes of COVID-19, including medical utilization and mortality, in both groups. However, pSS with HCQ group compared to pSS with MTX group subjects, pSS with HCQ had lower adverse outcomes (HR:0.878, 95% CI:0.697–1.105), but this was not statistically significant. Figure 3 shows Kaplan–Meier curves showing that subjects with pSS in the HCQ group did not have a significantly reduced risk compared with those in the MTX group (log-rank *p* = 0.267).

**Table 3.** Risk for adverse outcomes of patients with COVID-19.

Outcome	Patients with the outcome	Cumulative survival probability (%)				Hazard ratio* (95% CI)
		6 months	12 months	24 months	36 months	
Medical utilization						
Hospital inpatient services						
pSS treated with HCQ	116	95.69	93.13	89.21	85.97	0.812 (0.635, 1.039)
pSS treated with MTX	139	93.92	90.93	86.94	83.87	Reference
Critical care services, ICU						
pSS treated with HCQ	33	99.11	98.59	97.00	95.65	0.878 (0.549, 1.404)
pSS treated with MTX	37	99.03	98.09	97.04	95.04	Reference
Mechanical ventilation						
pSS treated with HCQ	10	99.80	99.59	99.31	98.72	0.685 (0.293, 1.603)
pSS treated with MTX	13	99.61	99.10	98.82	98.45	Reference
Mortality						
SS treated with HCQ	32	98.92	98.38	96.98	96.06	0.858 (0.535, 1.378)
SS treated with MTX	37	99.22	98.08	96.29	95.51	Reference
Adverse outcomes						
SS treated with HCQ	138	94.82	91.96	86.99	83.67	0.878 (0.697, 1.105)
SS treated with MTX	153	93.64	90.24	85.66	82.27	Reference

*Note*. Due to policy of TriNetX, any number less than 10 will be automatically assigned as <10. Adverse outcomes: Include medical utilization and mortality.

*Hazard ratio for outcomes among Sjögren’s syndrome patients in the HCQ group compared to Sjögren’s syndrome patients in the MTX group (after propensity score matching); *Propensity score matching (PSM) was performed on age at the index date, sex, race, socioeconomic status, lifestyle factors, comorbidities, and use of NSAIDs; *95% CI 95% confidence interval.

### Subgroup analyses

#### Sex

We further investigated the risk of COVID-19 incidence and adverse outcomes by dividing the study population into subgroups based on sex. There was no significant difference between the pSS patients in the HCQ cohort and the pSS patients in the MTX cohort (Supplementary Tables 2 and 3).

#### Age

We further investigated the risk of COVID-19 incidence and adverse outcomes by dividing the study population into subgroups based on their age at the index date (18–64 and over 65 years old) (Supplementary Tables 2 and 3). Among patients aged 18–64 years, pSS patients in the HCQ cohort had a significantly lower risk of contracting COVID-19 (HR:0.733, 95% CI: 0.561–0.960) than did pSS patients in the MTX cohort (Supplementary Table 2). There was no significant difference in the incidence of adverse outcomes of COVID-19 between the pSS patients in the HCQ cohort and the pSS patients in the MTX cohort (Supplementary Table 3).

#### Race

We then proceeded to perform a deeper investigation into different races (white, black, African American, or other races). There was no significant difference in the incidence of COVID-19 between the pSS patients in the HCQ cohort and the pSS patients in the MTX cohort among patients of different races (Supplementary Table 2). However, patients with pSS in the HCQ cohort had a significantly lower risk of adverse outcomes (HR: 0.410, 95% CI: 0.202–0.832) than did patients with pSS in the MTX cohort among patients of other races (Supplementary Table 3).

#### Comorbidity

There was no significant difference in the incidence of different comorbidities between the pSS patients in the HCQ cohort and the pSS patients in the MTX cohort (Supplementary Table 2). Regarding the comorbidities of CKD and neoplasms, the pSS patients in the HCQ cohort had a significantly lower risk of adverse outcomes (HR: 0.374, 95% CI: 0.161–0.873; HR: 0.658, 95% CI: 0.450–0.962, respectively) than did the pSS patients in the MTX cohort (Supplementary Table 3).

#### Medication

We further investigated the risk of COVID-19 incidence and adverse outcomes with corticosteroid treatment (Supplementary Tables 2 and 3). We did not find significant differences in corticosteroid use between the cohorts.

**Figure 4.**
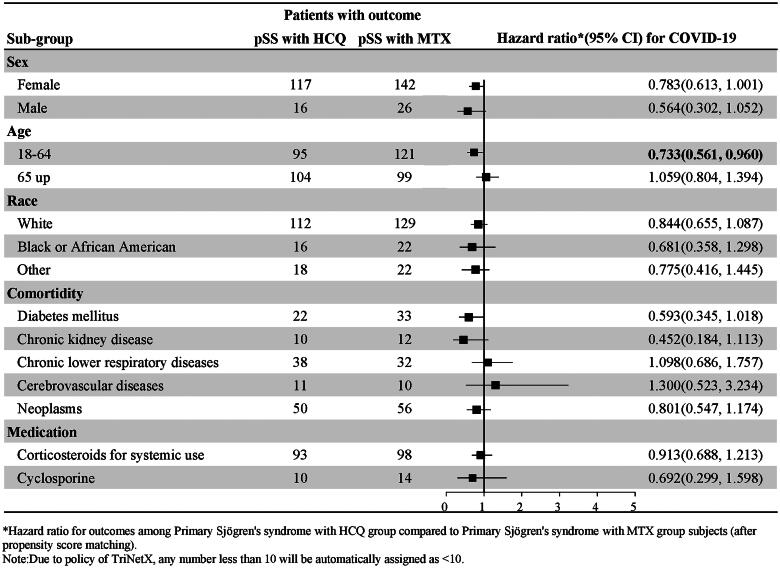
Subgroup analysis by Forest plots for the risk of COVID-19.

**Figure 5.**
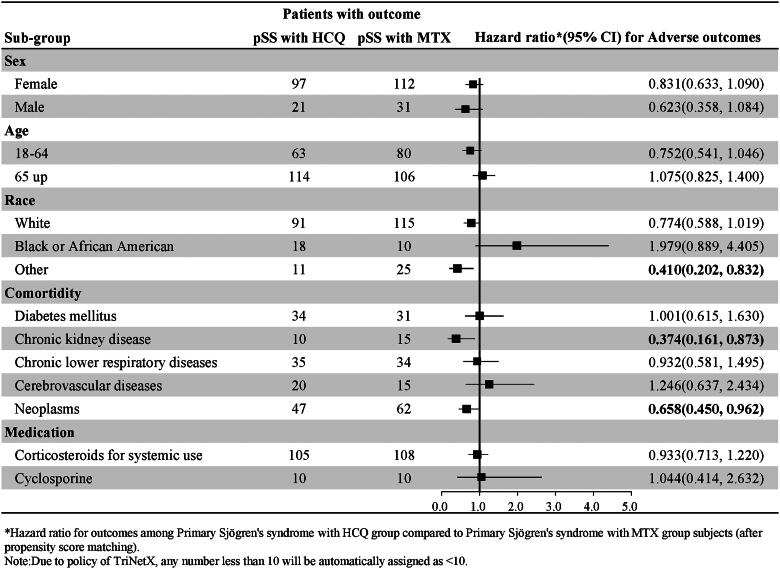
Subgroup analysis by Forest plots for the risk of adverse outcomes of COVID-19.

The above subgroup analyses for COVID-19 incidence and adverse outcomes are shown in the forest plots in Figures 4 and 5.

### Sensitivity analyses

We further conducted two sensitivity analyses to test the robustness of the results. For the pSS cohort without COVID-19 vaccination, compared to the pSS cohort treated with MTX, the pSS cohort treated with HCQ had a significantly lower risk of COVID-19 (positive laboratory test result or code) (HR:0.735, 95% CI 0.573–0.941) (Supplementary Table 4). There were no differences in medical utilization, mortality, or adverse outcomes between the cohorts. We modified the definition of the index date of pSS patients receiving HCQ or MTX for 3 months to 3 years. The pSS cohort treated with HCQ also had a significantly lower risk of COVID-19 (positive laboratory test result or code) (HR:0.732, 95% CI:0.574–0.933) (Supplementary Table 5). There were no differences in medical utilization, mortality, or adverse outcomes between the cohorts.

## Discussion

We retrieved data for pSS patients from 1 January 2020 and 30 June 2023, and our study revealed that the risk of COVID-19 (positive laboratory test result or code) was significantly lower in the pSS cohort treated with HCQ than in the pSS cohort treated with MTX (HR: 0.741, 95% CI 0.592–0.929). Kaplan–Meier curves are displayed as *p* = 0.009 according to the log-rank test. However, there were no significant differences in adverse outcomes (hospitalization, need for critical care services, need for mechanical ventilation, and mortality) of COVID-19 between the cohorts. Two sensitivity analyses also revealed similar findings in the pSS cohort without COVID-19 vaccination and in the pSS cohort receiving HCQ or MTX for 3 months to 3 years. Compared with the pSS cohort treated with MTX, the pSS cohort treated with HCQ had a significantly lower risk of COVID-19 (0.735, 0.573–0.941 and 0.732, 0.574–0.933, respectively).

According to the subgroup analyses, among the 18- to 64-year-old patients, those in the HCQ cohort had a significantly lower risk of COVID-19 (0.733, 0.61–0.960) than did those in the MTX cohort. The pSS patients in the HCQ cohort had a significantly lower risk of adverse outcomes (0.410, 0.202–0.873) than did those in the MTX cohort among patients of other races, excluding white, black, and African American individuals. Regarding the comorbidities of CKD and neoplasms, pSS patients in the HCQ cohort had a significantly lower risk of adverse outcomes (0.374, 0.161–0.873; 0.658, 0.450–0.962, respectively) than did pSS patients in the MTX cohort.

Despite the promising results obtained for HCQ activity against COVID-19 in 2020, the most recent outcomes highlighted the ineffectiveness of HCQ in the treatment of COVID-19. Several trials have shown that HCQ administration does not improve severe illness and does not prevent infection after SARS-CoV-2 exposure. In contrast, HCQ has arisen as a first-line treatment for managing autoimmune diseases such as RA, SLE, and SS. HCQ also improves glucose and lipid homeostasis and has significant antibacterial effects [23]. HCQ and MTX are the most commonly used csDMARDs [13]. HCQ is the immunomodulatory drug that is most often used to treat pSS [15]. From a pathophysiological point of view, HCQ interferes with Toll-like receptor signaling and inhibits the type I interferon pathway [24]. HCQ is usually prescribed in patients with fatigue, arthralgia, or myalgia rather than in those with severe systemic manifestations; however, it is known to have an effect on B-cell biomarkers, including decreasing IgG concentrations and the interferon signature [25]. In clinical practice, HCQ is used to treat conditions such as purpura (particularly when linked to hypergammaglobulinemia), cutaneous lesions, or inflammatory arthralgia.

Nayak et al. reported that MTX reduces immune activation due to off-target effects on the gut microbiota, potentially explaining the anti-inflammatory effects of MTX [26]. They revealed a mechanism of action of MTX and demonstrated that MTX targets the conserved purine and pyrimidine metabolic pathways that alter the gut microbiota community, leading to a decreased host immune response. The findings from this study have broad implications because, in addition to being given to patients with RA, MTX has been given to patients with inflammatory bowel diseases, multiple sclerosis, vasculitis, SLE, and other connective tissue diseases, transplantation, and cancer [27]. Habermann et al. showed that pausing MTX for one week prevented the impairment of Omicron BA.1 and BA.2 neutralization after COVID-19 booster vaccination [28]. Shirata et al. indicated that MTX attenuated immunogenicity following two doses of the SARS–CoV-2 mRNA vaccine in patients with RA, particularly resulting in dose-dependent suppression of the humoral immune response. Furthermore, MTX decreased the neutralizing activity against the Omicron variant, even after the third immunization [29].

In an Italian series of 164 patients with rheumatic autoimmune systemic diseases (RASDs), including 18 SS patients, the incidence of COVID-19 was significantly greater (*p* = 0.011) in RASD patients without ongoing csDMARD treatments, including HCQ, chloroquine, MTX, leflunomide, sulfasalazine, and cyclosporine [30]. Oztas et al. studied the frequency and severity of COVID-19 in patients with various rheumatic diseases, including 317 patients (197 with SLE, 79 with RA, and 41 with SS) treated regularly with colchicine or HCQ. Regular treatment with colchicine or HCQ did not result in the prevention of COVID-19 or the amelioration of its manifestations [16]. Guillaume et al. studied the prevalence of a history of COVID-19 infection among patients with SLE, RA, SS, or psoriatic arthritis (PsA) and the potential influence of long-term HCQ intake. They analyzed patients who did not receive HCQ (278 patients) and those who did receive HCQ (181 patients) among patients with different RASDs and found that DMARDs did not increase or decrease the risk of developing COVID-19 infection. The recruitment numbers were as follows: SS patients (HCQ: 50 patients, no HCQ: 114 patients), SLE (HCQ: 110 patients, no HCQ: 83 patients), RA (HCQ: 55 patients, no HCQ: 94 patients), and PsA (HCQ: 3 patients, no HCQ: 40 patients) [17].

Spila AS. et al. studied the risk of COVID-19 hospitalization and mortality in rheumatic patients treated with HCQ or other conventional DMARDs in Italy and found that exposure to HCQ/chloroquine was not associated with a protective effect against COVID-19-related outcomes. An increased risk for recent use of either MTX monotherapy (OR 1.19 [95% CI 1.05–1.34]) or other cDMARDs (OR 1.21 [95% CI 1.08–1.36]) compared with nonuse was found. The concomitant use of glucocorticoids and cDMARDs might increase the risk of COVID-19-related outcomes. However, their study included only patients with RA and SLE and did not include patients with pSS [18]. Alqatari et al. studied COVID-19 in patients with rheumatological diseases in the Eastern Province of Saudi Arabia and found that patients treated with HCQ and mycophenolate mofetil (MMF) for their underlying rheumatological diseases had a milder COVID-19 disease course. However, only patients with SLE and RA were enrolled [19].

Primary Sjögren’s syndrome is characterized by chronic activation of the innate immune system, particularly the type I interferon pathway, and persistent B-cell hyperactivity, leading to autoantibody production and glandular as well as systemic inflammation [7,12]. HCQ exerts its effects by interfering with endosomal acidification and TLR signaling, particularly TLR7 and TLR9, which are implicated in the aberrant activation of plasmacytoid dendritic cells and type I interferon production in pSS [24]. By modulating these pathways, HCQ may specifically counteract key pathogenic mechanisms in pSS, potentially reducing susceptibility to viral infections such as SARS-CoV-2 through dampening of inappropriate interferon-driven inflammation. In contrast, MTX primarily acts as an anti-metabolite, inhibiting folate-dependent enzymes and suppressing lymphocyte proliferation. While MTX is effective in reducing systemic inflammation, its broader immunosuppressive effects may impair host antiviral responses [18,26–29], which could be particularly relevant in the context of pSS, where immune dysregulation is already prominent. Thus, the intersection of pSS pathophysiology with these distinct mechanisms may partially explain the observed differences in COVID-19 risk between the two treatment groups in our study.

Compared with previous studies [16,17,30], our study using the TriNetX platform included significantly more pSS patients (2090 patients) treated with HCQ or MTX and found that the risk of COVID-19 in the pSS cohort treated with HCQ was 25.9% lower than that in the pSS cohort treated with MTX (HR: 0.741, 95% CI: 0.592–0.929). Our study also has certain limitations. First, we used validated outcome definitions and propensity score matching to avoid bias, but misclassification bias and residual confounding could not be completely avoided because of weaknesses inherent to studies using EMRs. Second, we did not include COVID-19 medication for analysis. Evolutionary effective treatments might affect COVID-19 outcomes. Third, TriNetX data come from hospital-based EMRs instead of population-based data. Therefore, vaccination can be speculated to be underreported. Fourth, more than 70% of the participants in our study were white Americans, and fewer than 5% were Asian; thus, the generalizability of our conclusions to non-white populations was limited. The epidemiology, clinical manifestations, and treatment responses of pSS as well as COVID-19 outcomes can vary by race, ethnicity, and healthcare access. Healthcare delivery systems and medication availability differ globally, which may impact the applicability of our results to non-white populations or those managed in different healthcare settings. Future studies including more diverse cohorts are warranted to validate and extend our findings.

## Conclusions

This is the first study to perform a head-to-head comparison of the risk and adverse outcomes of COVID-19 in a pSS cohort receiving HCQ and MTX. Compared with previous studies, our study from the TriNetX included a significant number of pSS patients (2090 patients) treated with HCQ or MTX. In this large, real-world cohort study, we found that pSS patients treated with hydroxychloroquine (HCQ) had a significantly lower risk of COVID-19 infection compared to those treated with methotrexate (MTX), although the risk of adverse COVID-19 outcomes did not differ significantly between the groups. The observed difference may be explained by the intersection of pSS pathophysiology and the distinct immunomodulatory mechanisms of these drugs. pSS is characterized by chronic activation of the type I interferon pathway and B-cell hyperactivity. HCQ, by interfering with endosomal acidification and TLR signaling, may specifically dampen the aberrant innate immune activation seen in pSS, potentially reducing susceptibility to viral infections such as SARS-CoV-2. In contrast, MTX broadly suppresses immune cell proliferation, which may impair antiviral defenses.

Clinically, these findings suggest that HCQ may be a preferable option for pSS patients at higher risk of COVID-19, particularly in the context of ongoing viral transmission. However, treatment decisions must remain individualized, considering disease activity, comorbidities, and patient preferences.

Our study has several limitations. The retrospective design and reliance on electronic health record data may introduce residual confounding and misclassification bias. The predominantly white American population in the TriNetX database may limit the generalizability of our results to more diverse populations and different healthcare settings. Additionally, we were unable to stratify patients by pSS disease severity or account for all potential confounders, such as medication adherence or over-the-counter drug use.

Future research should aim to validate these findings in prospective, multicenter, and more ethnically diverse cohorts. Further studies are also needed to explore the impact of pSS disease severity, other immunomodulatory therapies, and vaccination status on COVID-19 risk and outcomes in this population.

Our study provides important comparative data on the risk of COVID-19 in pSS patients treated with HCQ versus MTX, offering mechanistic and clinical insights that may inform therapeutic strategies during the ongoing pandemic.

## Supplementary Material

Supplementary materials0528.docx

## Data Availability

The data that support the findings of this study are available from the TriNetX Analytics Network (https://trinetx.com).
