# Supplementary material for: Comparative outcomes of COVID-19 in patients with primary Sjögren’s syndrome treated with hydroxychloroquine versus methotrexate: a retrospective cohort study
Source: Ann Med. 2025 Jul 6;57(1):2527361. doi: 10.1080/07853890.2025.2527361 (PMC12231326; doi:10.1080/07853890.2025.2527361)
Supplement: Supplementary materials0528.docx [file IANN_A_2527361_SM8083.docx]

**Supplementary materials**

**Table of contents**

Supplementary Table 1. Coding in this study **page 2**

Supplementary Table 2. Subgroup analysis of the incidence of COVID-19 **page 4**

Supplementary Table 3. Subgroup analysis of the adverse outcomes of patients with COVID-19 **page 5**

Supplementary Table 4. Sensitivity analysis of unvaccinated patients with COVID-19 **page 6**

Supplementary Table 5. Sensitivity analysis of patients with 3 months to 3 years of follow-up **page 7**

**Supplementary Table 1. Coding in this study**

| **Variable** | **Code(s)** |
| --- | --- |
| Study population |  |
| Sjögren's syndrome (SS) | ICD-10-CM: M35.0 |
| Exposed |  |
| Hydroxychloroquine | ATC code: P01BA02 |
| Methotrexate | ATC code: L01BA01, L04AX03 |
| Exclusion |  |
| Systemic lupus erythematosus (SLE) | ICD-10-CM:M32 |
| Rheumatoid arthritis (RA) | ICD-10-CM: M05, M06 |
| Scleroderma | ICD-10-CM: L90.0, L94.0, L94.1, L94.3 |
| Systemic sclerosis | ICD-10-CM: M34 |
| Dermatomyositis or Polymyositis | ICD-10-CM: M36.0, M33 |
| COVID-19 | Laboratory tests (result is positive), TNX code: 9088, LOINC: 94309-2, 94316-7, 94500-6, 94502-2, 94533-7, 94534-5, 94559-2, 94565-9, 94758-0, 94759-8, 94845-5, 95406-5, 95409-9, 95608-6, 96763-8, 94760-6  ICD-10-CM: U07.1, B34.2, J12.82, B97.29, Z86.16 |
| BMI | TNX code:9083 |
| Socioeconomic | ICD-10-CM: Z55-Z65 |
| Medical utilization |  |
| Office or Other Outpatient Services | CPT code:1013626 |
| Hospital Inpatient Services | CPT code:1013659 |
| Emergency Department Services | CPT code:1013711 |
| Lifestyle |  |
| Tobacco use | ICD-10-CM: Z72.0 |
| Nicotine dependence | ICD-10-CM: F17 |
| Alcohol related disorders | ICD-10-CM: F10 |
| Comorbidities |  |
| Hypertensive diseases | ICD-10-CM: I10-I1A |
| Diabetes mellitus | ICD-10-CM: E08-E13 |
| Hyperlipidemia | ICD-10-CM: E78 |
| Chronic kidney disease (CKD) | ICD-10-CM: N18 |
| Chronic lower respiratory diseases | ICD-10-CM: J40-J4A |
| Cerebrovascular diseases | ICD-10-CM: I60-I69 |
| Neoplasms | ICD-10-CM: C00-D49 |
| Noninfective enteritis and colitis | ICD-10-CM: K50-K52 |
| Diseases of liver | ICD-10-CM: K70-K77 |
| Sleep disorders | ICD-10-CM: G47 |
| Ischemic heart diseases | ICD-10-CM: I20-I25 |
| Depressive episode | ICD-10-CM: F32 |
| Medication |  |
| NSAIDs | ATC code: M01A |
| Corticosteroids for systemic use | ATC code: H02 |
| Abatacept | ATC code: L04AA24 |
| Rituximab | ATC code: L01FA01 |
| Sulfasalazine | ATC code: A07EC01 |
| Minocycline | ATC code: J01AA08 |
| Cyclophosphamide | ATC code: L01AA01 |
| Leflunomide | ATC code: L04AA13 |
| Azathioprine | ATC code: L04AX01 |
| Cyclosporine | ATC code: L04AA01 |
| Belimumab | RxNorm:1092437 |
| Tocilizumab | RxNorm:612865 |
| Ianalumab | RxNorm:OMOP5181464 |
| Outcome |  |
| COVID-19 | Laboratory tests (result is positive), TNX code: 9088, LOINC: 94309-2, 94316-7, 94500-6, 94502-2, 94533-7, 94534-5, 94559-2, 94565-9, 94758-0, 94759-8, 94845-5, 95406-5, 95409-9, 95608-6, 96763-8, 94760-6  ICD-10-CM: U07.1, B34.2, J12.82, B97.29, Z86.16 |
| Medical utilization |  |
| Hospital Inpatient Services | CPT code: 1013659, 1013699,1013729 |
| Critical Care Services, ICU | CPT code: 1013729 |
| Mechanical ventilation | CPT code:31500, 1015098, 1022227, ICD-10-PCS:5A1935Z, 5A1945Z, 5A1955Z, 0BH17EZ, 0BH18EZ, 0BH13EZ, or Extracorporeal membrane oxygenation, ECMO, ICD-9-CM:39.65 |
| Adverse outcomes | Include medical utilization and mortality |
| Sensitivity analysis |  |
| SARS coronavirus 2 vaccine | TNX code:213, CPT code:91303, 91302, 91305, 91306, 91307, 91300, 91301 |

Note:

TNX: TriNetX curated.

LOINC: Logical Observation Identifiers Names and Codes .

ICD-10-CM: International Classification of Diseases, Tenth Revision, Clinical Modification.

ATC: Anatomical Therapeutic Chemical Classification.

CPT: Current Procedural Terminology,

RxNorm: Normalized Drug Nomenclature

**Supplementary Table 2. Subgroup analysis of the incidence of COVID-19**

|  | **Patients in**  **cohort** | **Patients with outcome** | | **Hazard ratio***  **(95% CI)** |
| --- | --- | --- | --- | --- |
|  |  | **pSS with HCQ** | **pSS with MTX** |  |
| **Sex** |  |  |  |  |
| Female | 856 | 117 | 142 | 0.783(0.613, 1.001) |
| Male | 148 | 16 | 26 | 0.564(0.302, 1.052) |
| **Age** |  |  |  |  |
| 18-64 | 709 | 95 | 121 | **0.733(0.561, 0.960)** |
| 65up | 682 | 104 | 99 | 1.059(0.804, 1.394) |
| **Race** |  |  |  |  |
| White | 746 | 112 | 129 | 0.844(0.655, 1.087) |
| Black | 117 | 16 | 22 | 0.681(0.358, 1.298) |
| Other | 177 | 18 | 22 | 0.775(0.416, 1.445) |
| **Comorbidity** |  |  |  |  |
| Diabetes mellitus | 123 | 22 | 33 | 0.593(0.345, 1.018) |
| Chronic kidney disease (CKD) | 35 | 10 | 12 | 0.452(0.184, 1.113) |
| Chronic lower respiratory diseases | 127 | 38 | 32 | 1.098(0.686, 1.757) |
| Cerebrovascular diseases | 46 | 11 | 10 | 1.300(0.523, 3.234) |
| Neoplasms | 229 | 50 | 56 | 0.801(0.547, 1.174) |
| **Medication** |  |  |  |  |
| Corticosteroids for systemic use | 644 | 93 | 98 | 0.913(0.688, 1.213) |

Note:

Bold font indicates statistical significance.

*Hazard ratio for outcomes among Sjögren's syndrome with HCQ group compared to Sjögren's syndrome with MTX group subjects (after propensity score matching).

95% CI, 95% confidence interval.

**Supplementary Table 3. Subgroup analysis of the adverse outcomes of patients with COVID-19**

|  | **Patients in**  **cohort** | **Patients with outcome** | | **Hazard ratio***  **(95% CI)** |
| --- | --- | --- | --- | --- |
|  |  | **pSS with HCQ** | **pSS with MTX** |  |
| **Sex** |  |  |  |  |
| Female | 856 | 97 | 112 | 0.831(0.633, 1.090) |
| Male | 148 | 21 | 31 | 0.623(0.358, 1.084) |
| **Age** |  |  |  |  |
| 18-64 | 709 | 63 | 80 | 0.752(0.541, 1.046) |
| 65up | 682 | 114 | 106 | 1.075(0.825, 1.400) |
| **Race** |  |  |  |  |
| White | 746 | 91 | 115 | 0.774(0.588, 1.019) |
| Black | 117 | 18 | 10 | 1.979(0.889, 4.405) |
| Others | 177 | 11 | 25 | **0.410(0.202, 0.832)** |
| **Comorbidity** |  |  |  |  |
| Diabetes mellitus | 123 | 34 | 31 | 1.001(0.615, 1.630) |
| Chronic kidney disease (CKD) | 35 | 10 | 15 | **0.374(0.161, 0.873)** |
| Chronic lower respiratory diseases | 127 | 35 | 34 | 0.932(0.581, 1.495) |
| Cerebrovascular diseases | 46 | 20 | 15 | 1.246(0.637, 2.434) |
| Neoplasms | 229 | 47 | 62 | **0.658(0.450, 0.962)** |
| **Medication** |  |  |  |  |
| Corticosteroids for systemic use | 644 | 105 | 108 | 0.933(0.713, 1.220) |

Note:

Bold font indicates statistical significance.

*Hazard ratio for outcomes among Sjögren's syndrome with HCQ group compared to Sjögren's syndrome with MTX group subjects (after propensity score matching).

95% CI, 95% confidence interval.

**Supplementary Table 4. Sensitivity analysis of unvaccinated patients with COVID-19**

| **Outcome** | **Patients with outcome** | | **Hazard ratio***  **(95% CI)** |
| --- | --- | --- | --- |
|  | **pSS with HCQ** | **pSS with MTX** |  |
| Incidence of COVID-19 |  |  |  |
| COVID-19 (Laboratory tests: positive) | 38 | 48 | 0.768(0.502, 1.176) |
| COVID-19 (ICD-10-CM Code) | 102 | 131 | **0.744(0.575, 0.964)** |
| COVID-19 (Positive or ICD-10-CM Code) | 111 | 144 | **0.735(0.573, 0.941)** |
| Medical utilization |  |  |  |
| Hospital Inpatient Services | 100 | 107 | 0.906(0.690, 1.190) |
| Critical Care Services, ICU | 16 | 25 | 0.619(0.331, 1.160) |
| Mechanical ventilation | 10 | 10 | 0.765(0.285, 2.055) |
| Mortality | 23 | 36 | 0.625(0.370, 1.055) |
| Adverse outcomes | 112 | 119 | 0.912(0.704, 1.180) |

Note: Due to policy of TriNetX, any number less than 10 will be automatically assigned as <10.

Bold font indicates statistical significance.

*Hazard ratio for outcomes among Sjögren's syndrome with HCQ group compared to Sjögren's syndrome with MTX group subjects (after propensity score matching).

95% CI, 95% confidence interval.

**Supplementary Table 5. Sensitivity analysis of patients with 3 months to 3 years of follow-up**

| **Outcome** | **Patients with outcome** | | **Hazard ratio***  **(95% CI)** |
| --- | --- | --- | --- |
|  | **pSS with HCQ** | **pSS with MTX** |  |
| Incidence of COVID-19 |  |  |  |
| COVID-19 (Laboratory tests: positive) | 41 | 52 | 0.752(0.500, 1.133) |
| COVID-19 (ICD-10-CM code) | 102 | 133 | **0.720(0.557, 0.932)** |
| COVID-19 (Positive or ICD-10-CM Code) | 116 | 149 | **0.732(0.574, 0.933)** |
| Medical utilization |  |  |  |
| Hospital Inpatient Services | 92 | 109 | 0.809(0.613, 1.068) |
| Critical Care Services, ICU | 23 | 29 | 0.758(0.438, 1.310) |
| Mechanical ventilation | 10 | 10 | 0.605(0.198, 1.849) |
| Mortality | 25 | 33 | 0.727(0.433, 1.223) |
| Adverse outcomes | 107 | 120 | 0.854(0.658, 1.108) |

Note: Due to policy of TriNetX, any number less than 10 will be automatically assigned as <10.

Bold font indicates statistical significance.

*Hazard ratio for outcomes among Sjögren's syndrome with HCQ group compared to Sjögren's syndrome with MTX group subjects (after propensity score matching).

*95% CI, 95% confidence interval
